# Risk assessment of post‐myocardial infarction patients with preserved ejection fraction using 45‐min short resting Holter electrocardiographic recordings

**DOI:** 10.1111/anec.13087

**Published:** 2023-09-12

**Authors:** Konstantinos Triantafyllou, Nikolaos Fragakis, Konstantinos A. Gatzoulis, Antonios Antoniadis, Georgios Giannopoulos, Petros Arsenos, Dimitrios Tsiachris, Christos‐Konstantinos Antoniou, Konstantinos Trachanas, Konstantinos Tsimos, Vassilios Vassilikos

**Affiliations:** ^1^ Third Cardiology Department, Hippokration Hospital Aristotle University of Thessaloniki Thessaloniki Greece; ^2^ First Department of Cardiology, Hippokration General Hospital National and Kapodistrian University of Athens School of Medicine Athens Greece; ^3^ State Department of Cardiology Hippokration General Hospital Athens Greece; ^4^ Department of Cardiology, Faculty of Medicine University of Ioannina Ioannina Greece

**Keywords:** electrocardiography, heart rate variability, Holter, ischaemic heart disease, noninvasive risk factors, risk stratification

## Abstract

**Background:**

Risk stratification for sudden cardiac death in post‐myocardial infarction (post‐MI) patients remains a challenging task. Several electrocardiographic noninvasive risk factors (NIRFs) have been associated with adverse outcomes and were used to refine risk assessment. This study aimed to evaluate the performance of NIRFs extracted from 45‐min short resting Holter ECG recordings (SHR), in predicting ventricular tachycardia inducibility with programmed ventricular stimulation (PVS) in post‐MI patients with preserved left ventricular ejection fraction (LVEF).

**Methods:**

We studied 99 post‐MI ischemia‐free patients (mean age: 60.5 ± 9.5 years, 86.9% men) with LVEF ≥40%, at least 40 days after revascularization. All the patients underwent PVS and a high‐resolution SHR. The following parameters were evaluated: mean heart rate, ventricular arrhythmias (premature ventricular complexes, couplets, tachycardias), QTc duration, heart rate variability (HRV), deceleration capacity, heart rate turbulence, late potentials, and T‐wave alternans.

**Results:**

PVS was positive in 24 patients (24.2%). HRV, assessed by the standard deviation of normal‐to‐normal R–R intervals (SDNN), was significantly decreased in the positive PVS group (42 ms vs. 51 ms, *p* = .039). SDNN values <50 ms were also associated with PVS inducibility (OR 3.081, *p* = .032 in univariate analysis, and 4.588, *p* = .013 in multivariate analysis). No significant differences were identified for the other NIRFs. The presence of diabetes, history of ST‐elevation MI (STEMI) and LVEF <50% were also important predictors of positive PVS.

**Conclusions:**

HRV assessed from SHR, combined with other noninvasive clinical and echocardiographic variables (diabetes, STEMI history, LVEF), can provide an initial, practical, and rapid screening tool for arrhythmic risk assessment in post‐MI patients with preserved LVEF.

## Graphical abstract

This study evaluates the performance of various clinical parameters and NIRFs extracted from short‐duration ECG recordings, in predicting ventricular tachycardia inducibility with PVS, in post‐MI patients with preserved LVEF. We found that reduced HRV, presence of diabetes, history of STEMI and LVEF <50% were significant predictors of positive PVS.

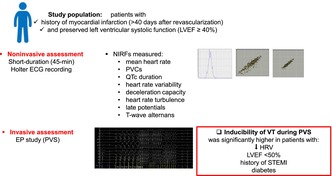

## 1 INTRODUCTION

Post‐MI patients are at increased risk of sudden cardiac death (SCD) and their management remains a challenge. The indication for implantable cardioverter‐defibrillator (ICD) implantation for primary prevention is currently solely based on left ventricular ejection fraction (LVEF) ≤ 35% (Al‐Khatib et al., 2018; Zeppenfeld et al., 2022). However, approximately 85% of post‐MI patients maintain a preserved or mildly reduced LVEF (≥40%), mainly due to the wide implementation of primary percutaneous coronary intervention in acute events. Remarkably, no preventive measures are recommended for these patients, even though their annual SCD incidence is estimated at 1% (Pannone et al., 2021).

Several noninvasive electrocardiographic (ECG) parameters, such as heart rate (HR), QRS duration, ectopic ventricular activity, late potentials (LPs), repolarization abnormalities and indices of the cardiac autonomic nervous system, have been studied as potentially useful tools to identify high‐risk asymptomatic post‐MI patients with preserved left ventricular systolic function. These parameters may indicate an arrhythmic substrate, reflecting different patterns of arrhythmogenicity, i.e., the presence of fibrotic, arrhythmogenic areas (ventricular ectopic activity, LPs), abnormal ventricular repolarization [QTc, T‐wave alternans (TWAs)] and autonomic nervous system dysfunction [heart rate variability (HRV), deceleration capacity (DC), heart rate turbulence (HRT)] (Wellens et al., 2014). However, the specific value of the above parameters remains unclear.

The role of PVS has been established as a risk stratification tool for post‐MI patients, across the whole spectrum of left ventricular systolic function (Arsenos et al., 2020; Schmitt et al., 2001), even in cases with preserved LVEF (Gatzoulis et al., 2013, 2014). Interestingly, in a prospective study, positive PVS had a 33% predictive power to identify post‐MI patients who developed malignant ventricular arrhythmias during follow‐up. In contrast, a negative electrophysiological study indicates less than 3% chance of developing SCD two years after the index event, irrespective of LVEF (Schmitt et al., 2001). Moreover, positive PVS was found to be a more accurate predictor of major arrhythmic events (fatal or life‐threating arrhythmias) in post‐MI patients compared with reduced LVEF (odds ratio 8.5 vs. 5.1, respectively) (Bailey et al., 2001).

The combination of invasive PVS with noninvasive ECG parameters has also been suggested in multivariable prognostic models as a more accurate risk assessment tool (Arsenos et al., 2020; Dagres & Hindricks, 2013; de Ferrari et al., 2007; Deyell et al., 2015). These noninvasive ECG indices are routinely measured in long‐duration (usually 24 h) ambulatory Holter ECG recordings. However, short‐duration ECG recordings, ranging from 5 min to a few hours, have also been used, with promising results (Arsenos et al., 2018, 2022; Berkowitsch et al., 2004; Couderc et al., 2007; La Rovere et al., 2003; Mewton et al., 2016; Rizas et al., 2018).

The concept of using short‐duration ECG Holter recordings (SHRs) for the assessment of noninvasive ECG risk parameters, and consequently SCD risk stratification, appears attractive. The development of a fast, practical, and effective risk evaluation method, which could be applied even during a single outpatient clinic visit, would be beneficial for both patients and physicians. This study aims to assess the performance of noninvasive ECG parameters, extracted from 45‐min resting SHR, in distinguishing the subset of post‐MI patients who are potentially at high risk of SCD as guided by a positive PVS.

## 2 METHODS

The study protocol was approved by the Institutional Ethics Committees and all patients provided written informed consent. The study population consisted of post‐MI patients, enrolled in the PRESERVE‐EF study, a multicentre, prospective, observational cohort study, in 4 tertiary cardiology departments (Gatzoulis et al., 2019). Briefly, in this study, seven ECG noninvasive risk factors (NIRFs) extracted from 24‐h Holter ECG recordings [i.e. LPs, premature ventricular complexes (PVCs), presence of non‐sustained ventricular tachycardia (NSVT) episode, HRV, QTc duration, ambulatory TWA, and combined DC, and HRT] were evaluated in post‐MI patients with LVEF ≥40%, on optimal tolerated medical therapy, at least 40 days after the acute ischemic event, provided that any active myocardial ischemia had been excluded. Patients with (i) active residual myocardial ischemia, (ii) presence of a secondary prevention indication for ICD implantation, (iii) permanent pacemaker, (iv) persistent, or permanent atrial fibrillation, (v) neurological symptoms (presyncope or syncope) within the last 6 months, (vi) systemic illness (cancer, liver failure, end‐stage renal disease, and thyroid dysfunction), (vii) antiarrhythmic medication other than b‐blockers, and (viii) age >80 or <18 years, were not included. In the presence of at least one abnormal NIRF in 24‐h Holter ECG monitoring, patients underwent invasive PVS. The electrophysiological study was performed according to the following protocol: Stimuli were introduced in 2 right ventricular sites (apex and outflow tract) at two drive train cycle lengths (550 and 400 ms) at each site. Up to 3 extrasystoles were introduced after the drive train at each drive train cycle length with coupling intervals progressively (10 ms increments) shortened down to 200 ms or until refractoriness was reached, starting from the last extrasystole. Patients were classified as inducible, if sustained monomorphic ventricular tachycardia (VT), ventricular flutter or polymorphic VT degenerated into ventricular fibrillation were induced, and in these patients, an ICD was implanted.

In our analysis, we focused on patients with abnormal 24‐h NIRFs as part of the PRESERVE‐EF study, who underwent PVS. All the participants had a 45‐min high‐resolution SHR, in the supine position, at rest. Recordings were performed in the morning or early afternoon hours, i.e., between 8:00 am and 2:00 pm, in order to eliminate circadian variability of the measured NIRFs. CardioMem CM 4000 Multi‐channel ECG Recorder (GE Healthcare—GETEMED), with three *x*, *y*, *z* orthogonal, bipolar leads, was used for recording. Data were analyzed with CardioDay v.2.4 software (GE Healthcare). Correction of automatic measurements and exclusion of artifacts was performed after a careful review of each recording. We studied the following parameters: mean HR, PVCs, ventricular couplets, VT, HRV, DC, HRT onset and slope, TWA, LPs, and mean QTc duration. Each parameter was analyzed as a numeric variable, as well as a dichotomous variable, using normal cut‐off points suggested by relevant previous publications (Berkowitsch et al., 2004; Yodogawa & Shimizu, 2014). Specifically, TWA was measured by the modified moving average (MMA) method, with the analysis of three channel records. In our study, the greater maximal voltage of MMA–TWA was chosen for assessment. MMA–TWA was determined as positive if the voltage was >60 μV in at least 2 leads (Couderc et al., 2007; Minkkinen et al., 2009; Verrier et al., 2011). HRV was assessed with the time‐domain method, measuring the standard deviation of normal‐to‐normal R‐R intervals (SDNN). Abnormal values for SDNN were defined as <50 ms (Shaffer & Ginsberg, 2017; Task Force of the European Society of Cardiology and the North American Society of Pacing and Electrophysiology, 1996). DC measurement was based on the phase‐rectified signal averaging method, which is a specific signal‐processing algorithm capable of extracting periodic components out of non‐stationary biological signals. DC was considered abnormal for values ≤2.5 ms (Hamm et al., 2017; Rizas et al., 2018). HRT was measured automatically using an algorithm applied to the Holter software. HRT parameters included turbulence onset (TO) and turbulence slope (TS). The presence of PVCs was necessary for HRT calculations. Abnormal values for HRT are defined as ≥0% for TO, and ≤2.5 ms/RR interval for TS (Bauer et al., 2008; Berkowitsch et al., 2004). The automatic measurement of QT duration was performed every 30 averaged beats. Fridericia's formula was selected for the correction of QT interval measurements since it shows the best correction rate and significantly improves the prediction of short‐ and long‐term mortality (Vandenberk et al., 2016). QTc interval was considered increased for values >440 ms in men and >450 ms in women (Arsenos et al., 2022; Gatzoulis et al., 2019). Finally, the following criteria were used for the detection of LPs: filtered QRS complex duration ≥114 ms, root mean square voltage of the terminal 40 ms of the filtered QRS (RMS‐40) < 20 μV, and duration of the terminal portion of the QRS complex that display less than 40 μV amplitude signals (LAS‐40) > 38 ms (Breithardt et al., 1991; Goldberger et al., 2008).

Clinical evaluation included medical history, demographic parameters, and laboratory blood tests. A resting 12‐lead surface ECG in the supine position and sinus rhythm was obtained at a paper speed of 25 mm/s (sensitivity 10 mV/cm). We evaluated the P wave, PR interval, QRS complex, QT interval, and RR duration. Fridericia formula was used for the correction of the QT interval measurements. The echocardiographic examination included LVEF estimation using Simpson's biplane method, left ventricular end‐diastolic diameter, left ventricular wall thickness of the interventricular septum, and the posterior wall and left atrial diameters using the M‐mode technique in the parasternal long‐axis view.

### 2.1 Statistical analysis

Data were analyzed using the SPSS Statistics version 27 software (IBM Corp.). Continuous variables are presented as mean ± standard deviation or median [interquartile range] if their values were not normally distributed. The examination of normality was performed by the Kolmogorov–Smirnov test. Comparisons of the continuous variables were performed using the Student's *t*‐test or the non‐parametric Mood's median test and Mann–Whitney *U*‐test, as appropriate. The categorical variables are expressed as absolute numbers and frequencies and were compared using Pearson's chi‐squared test or 2‐tail Fisher's exact test. Binary logistic regression analysis was conducted to estimate the odds ratio (OR) for the measured variables between groups regarding inducibility in PVS. We calculated the sensitivity, specificity, and predictive values of variables that were different between PVS positive and PVS negative groups. Moreover, receiver operating characteristic (ROC) analysis was conducted and the area under the ROC curve (AUC) was interpreted as the probability of a positive PVS. A level of significance of <0.05 was used in all instances.

## 3 RESULTS

The study population consisted of 99 patients. Of these, 24.2% had positive PVS and 75.8% had negative PVS. Table 1 presents the demographic, clinical, and laboratory data of all participants as well as those with positive and negative PVS. All variables were similar between the groups, except for diabetes and type of infarction. Diabetes was more prevalent in the high‐risk group (41.7% vs.16%, *p* = .008), as well as the history of ST‐elevation MI (STEMI) (83.3% vs. 56%, *p* = .016). In univariate analysis, the OR was estimated 3.750 (95% CI 1.353–10.396, *p* = .011) and 3.929 (95% CI 1.224–12.611, *p* = .021) for positive PVS, for the above variables, respectively.

**TABLE 1:** Demographic and clinical characteristics of the study population.

Variable		All (*N* = 99)	− PVS (*N* = 75)	+ PVS (*N* = 24)	*p*
Age (years)		60.5 ± 9.5	59.8 ± 9.7	62.9 ± 8.7	.216
Gender (male)		86 (86.9)	63 (84)	23 (95.8)	.135
BMI (kg/m^2^)		27.1 [24.8, 30.4]	27.7 [25.1, 30.8]	25.8 [24.3, 29.2]	.378
Diabetes		22 (22.2)	12 (16)	10 (41.7)	.008**
Hypertension		60 (60.6)	42 (56)	18 (75)	.097
Dyslipidemia		61 (61.6)	43 (57.3)	18 (75)	.121
Smoking		41 (41.4)	32 (42.7)	9 (37.5)	.655
Alcohol consumption	No	62 (62.6)	43 (57.3)	19 (79.2)	.149
	Moderate	36 (36.4)	31 (41.3)	5 (20.8)	
	Excessive	1 (1)	1 (1.3)	0 (0)	
Coffee consumption	No	12 (12.1)	9 (12)	3 (12.5)	.430
	Moderate	82 (82.8)	61 (81.3)	21 (87.5)	
	Excessive	5 (5.1)	5 (6.7)	0 (0)	
Hereditary CAD		19 (19.2)	11 (14.7)	8 (33.3)	.071
Hereditary SCD		6 (6.1)	5 (6.7)	1 (4.2)	.655
Type of infarction	STEMI	62 (62.6)	42 (56)	20 (83.3)	.016*
	NSTEMI	37 (37.4)	33 (44)	4 (16.7)	
Reperfusion strategy	None	11 (11.1)	9 (12)	2 (8.3)	.207
	PCI	72 (72.7)	57 (76)	15 (62.5)	
	CABG	10 (10.1)	5 (6.7)	5 (20.8)	
	PCI + CABG	6 (6.1)	4 (5.3)	2 (8.3)	
Number of arteries with CAD	1	60 (60.6)	49 (65.3)	11 (45.8)	.228
	2	23 (23.2)	15 (20)	8 (33.3)	
	3	16 (16.2)	11 (14.7)	5 (20.8)	
LAD		57 (57.6)	41 (54.7)	16 (66.7)	.301
LCx		43 (43.4)	32 (42.7)	11 (45.8)	.785
RCA		56 (56.6)	41 (54.7)	15 (62.5)	.500
NYHA Class I		82 (82.8)	64 (85.3)	18 (75)	.243
Hemoglobin (g/dL)		14.1 ± 1.2	14 ± 1.1	14.1 ± 1.3	.670
Glucose (mg/dL)		100 [91, 124.5]	99 [91, 110.5]	120 [93, 141]	.168
BUN (mg/dL)		34.4 ± 16.7	32.7 ± 14.7	39.1 ± 21	.280
Creatinine (mg/dL)		0.95 [0.82, 1.10]	0.94 [0.80, 1.01]	1 [0.89, 1.15]	.549
Uric acid		5.8 [4.9, 6.8]	5.6 [4.6, 6.8]	6.3 [5.4, 6.7]	.160
Potassium (mmol/L)		4.3 [4.1, 4.6]	4.2 [4.0, 4.5]	4.4 [4.1, 4.6]	.160
Sodium (mmol/L)		139 [138, 141]	139 [138, 142]	139 [136, 140]	.590
ALT (U/L)		24 [16.5, 37]	24 [16, 33.5]	28 [17, 42]	.511
LDL (mg/dL)		101.9 ± 33.8	104.1 ± 33.3	96.1 ± 35.1	.350
HDL (mg/dL)		39.5 ± 9.6	39.6 ± 10.2	39.3 ± 7.8	.996
Triglycerides (mg/dL)		130.5 ± 44.5	127.9 ± 47	137.6 ± 36.8	.255
Aspirin		94 (94.9)	72 (96)	22 (91.7)	.592
P2Y12 inhibitor		85 (85.9)	67 (89.3)	18 (75)	.097
Statin		95 (96)	71 (94.7)	24 (100)	.569
B‐blocker		90 (90.9)	67 (89.3)	23 (95.8)	.448
ACEi or ARB		72 (72.7)	53 (70.7)	19 (79.2)	.416

*Note*: Values are shown as mean ± SD, median [interquartile range] and *N* (%).

Abbreviations: ACEi, angiotensin‐converting enzyme inhibitor; ALT, alanine transaminase; ARB, angiotensin receptor blocker; BMI, body mass index; BUN, Blood urea nitrogen; CABG, coronary artery bypass grafting; CAD, coronary artery disease; HDL, high‐density cholesterol; LAD, left anterior descending artery; LCx, left circumflex artery; LDL, low‐density cholesterol; NSTEMI, non ST‐elevation myocardial infarction; NYHA, New York Heart Association; PCI, percutaneous coronary intervention; PVS, programmed ventricular stimulation; RCA, right coronary artery; SCD. sudden cardiac death; STEMI, ST‐elevation myocardial infarction.

*p < .05; **p < .01.

The echocardiographic and baseline 12‐lead electrocardiographic parameters are demonstrated in Table 2. There was no statistically significant difference in the electrocardiographic measurements. Patients with a positive PVS had increased left ventricular end‐diastolic diameter (53.7 ± 7.1 mm vs. 50.2 ± 6.1 mm, *p* = .028) and reduced LVEF (45% vs. 50%, *p* < .001), compared with the PVS negative group. Importantly, LVEF less than 50% yielded an OR of 4.759 (95% CI 1.692–13.386, *p* = .003) for PVS inducibility.

**TABLE 2:** Electrocardiographic and echocardiographic parameters of the groups.

	− PVS	+ PVS	*p*
12‐lead ECG			
P (ms)	110 [90, 120]	93.6 [76, 115.5]	.096
PR (ms)	160 [142, 180.5]	160 [149.5, 200]	.725
QRS (ms)	96 [87.5, 102]	90 [85, 100]	.509
QRS >120 ms	8 (10.7)	1 (4.2)	.448
QTc (ms)	420 ± 30.6	414.9 ± 44	.711
RR (ms)	963.8 ± 136.5	986.7 ± 156.4	.410
Transthoracic Echocardiogram			
LVEF (%)	50 [45, 55]	45 [40, 49]	<.001**
LVEF <50%	29 (38.7)	18 (75)	.002**
LVEDD (mm)	50.2 ± 6.1	53.7 ± 7.1	.028*
LV wall thickness (mm)	9.9 ± 1.5	10.1 ± 1.4	.469
LA diameter (mm)	40.1 ± 4.6	41.4 ± 4.5	.227

*Note*: Values are shown as mean ± SD, median [interquartile range] and *N* (%).

Abbreviations: ECG, electrocardiogram; LA, left atrium; LV, left ventricle; LVEDD, left ventricular end diastolic diameter; LVEF, left ventricular ejection fraction.

*p < .05; **p < .01.

**FIGURE 1:**
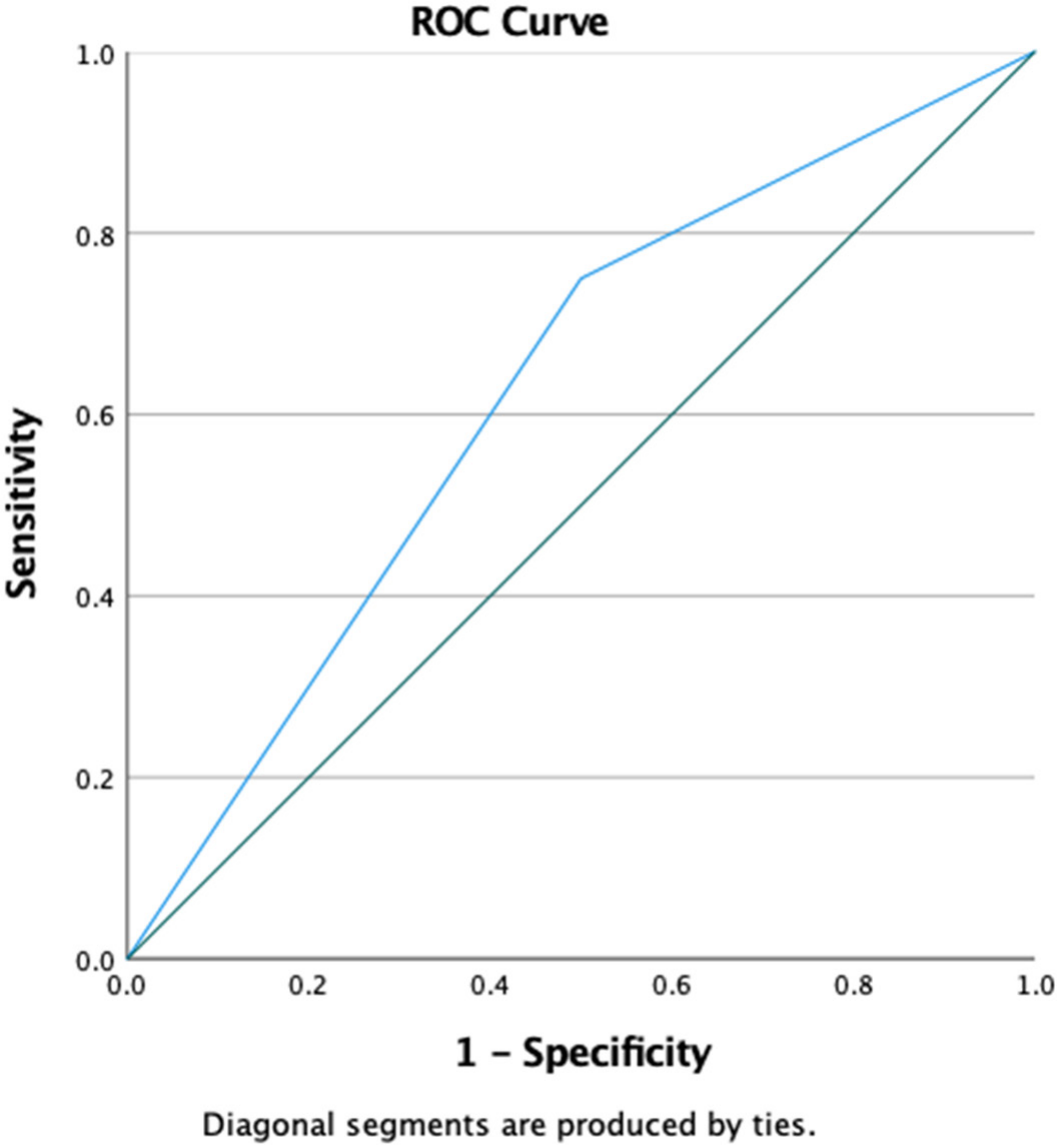
Receiver operating characteristic (ROC) curve of the standard deviation of normal‐to‐normal R–R intervals (SDNN) variable for predicting positive Programmed Ventricular Stimulation.

Data extracted from SHR are summarized in Table 3. HRV was the only noninvasive index that was significantly different between the two groups. Patients with a positive PVS had a higher prevalence of abnormal HRV, assessed as a dichotomous variable (75% vs. 50%, *p* = .028). The estimated sensitivity, specificity, positive predictive value, negative predictive value, and accuracy of abnormal HRV in predicting a positive PVS were 75%, 50.7%, 65.5%, 61.9%, and 56.6%, respectively, and the AUC was 0.625 (Figure 1). In univariate analysis, the OR for abnormal HRV as a predictor of a positive PVS was 3.08 (95% CI 1.10–8.62, *p* = .032).

**TABLE 3:** Short‐duration Holter parameters of the groups.

Holter parameter	− PVS	+ PVS	*p*
Mean HR (bpm)	63.3 ± 8.1	62.2 ± 7.9	.457
PVCs	2 [0, 26]	2.5 [0, 11]	.690
PVCs > 13	24 (32)	5 (20.8)	.295
Presence of V Couplets or NSVT	9 (12)	2 (8.3)	.619
SAECG fQRSd (ms)	98 [92, 115]	105 [95.3, 116.5]	.323
LAS (ms)	34 [28, 40]	33 [22.3, 45.8]	.990
RMS (μV)	30 [14.4, 57.8]	23.6 [13.6, 45.9]	.519
SAECG fQRSd > 114 ms	17 (22.7)	7 (29.2)	.518
LAS > 38 ms	24 (32)	10 (41.7)	.385
RMS < 20 μV	27 (36)	10 (41.7)	.617
Presence of LPs (≥2 abnormal SAECG parameters)	24 (32)	10 (41.7)	.385
SDNN (ms)	51 [35, 66]	42 [37.5, 58]	.039*
SDNN < 50 ms	37 (50)	18 (75)	.028*
DC	5.5 [2.6, 7.5]	5.1 [3.1, 8]	.723
DC ≤ 2.5	18 (24)	4 (16.7)	.452
TWA	26 [17, 37]	23.5 [15, 47]	.513
TWA > 60	3 (4)	4 (16.7)	.057
HRT onset	−0.02 [−0.05, −0.01]	−0.03 [−0.05, −0.02]	.117
HRT slope	11.9 [6.3, 16.5]	10.4 [7.7, 19.1]	.792
≥1 abnormal HRT parameters	8 (10.7)	0 (0)	.193
QTc duration (ms)	440 ± 26.6	435 ± 21.3	.570
Increased QTc (M > 440 ms, F > 450 ms)	34 (45.3)	10 (41.7)	.753

*Note*: Values are shown as mean ± SD, median [interquartile range] and *N* (%).

Abbreviations: DC, deceleration capacity; F, female; fQRSd, filtered QRS duration; HR, heart rate; HRT, heart rate turbulence; HRV, heart rate variability; LAS, low amplitude signal (<40 μV) duration in the terminal filtered QRS; LPs, late potentials; M, male; NSVT, non‐sustained ventricular tachycardia; PVC, premature ventricular complex; PVS, programmed ventricular stimulation; RMS, root mean square voltage of the terminal 40 ms of the filtered QRS; SAECG, signal‐averaged ECG; SDNN, standard deviation of normal‐to‐normal R‐R intervals; TWA; T‐wave alternans; V, ventricular.

*p < .05.

Based on the previous findings, the presence of diabetes, history of STEMI, LVEF, and SDNN were considered for multivariate logistic regression analysis (Table 4). The estimated risk for PVS inducibility ranged between 4 and 5, for all variables. Specifically, the OR for SDNN <50 ms was 4.588 (95% CI 1.378–15.275, *p* = .013), comparable to LVEF.

**TABLE 4:** Logistic regression analysis of the predictors for PVS inducibility.

Variable	Univariate analysis			Multivariate analysis		
	OR	95% CI	*p*	OR	95% CI	*p*
Diabetes	3.750	1.353–10.396	.011	3.970	1.162–13.565	.028
Infarction type (STEMI)	3.929	1.224–12.611	.021	4.888	1.259–18.980	.022
LVEF <50%	4.759	1.692–13.386	.003	4.999	1.570–15.919	.006
SDNN <50 ms	3.081	1.101–8.621	.032	4.588	1.378–15.275	.013

Abbreviaitons: LVEF, left ventricular ejection fraction; SDNN, standard deviation of normal‐to‐normal R‐R intervals; STEMI, ST‐elevation myocardial infarction.

## 4 DISCUSSION

The purpose of this study was to investigate the value of NIRFs, extracted from SHR, in predicting the outcome of PVS in patients with post‐MI with preserved LVEF. The main finding of the study is that reduced HRV is a significant, independent risk marker of positive PVS, which may have important clinical implications. The other NIRFs were not different between the compared groups.

The selected ECG NIRFs have been extensively studied as indices of post‐infarction fibrosis (PVCs, NSVT, LPs), cardiac repolarization abnormalities (TWA, QTc), and impaired autonomic nervous system function (HR, HRV, DC, HRT) (Wellens et al., 2014). Many studies have shown that post‐MI patients with LVEF ≥35% with abnormal NIRFs are at increased risk for SCD (Bauer et al., 2009; Hashimoto et al., 2021; Ikeda et al., 2006; La Rovere et al., 1998; Schmidt et al., 1999; Vaage‐Nilsen et al., 1998). Noninvasive ECG parameters are usually assessed in long‐term, mainly 24‐h recordings. However, data supporting the prognostic role of SHR also exist (Arsenos et al., 2018, 2022; Berkowitsch et al., 2004; Rizas et al., 2018). We decided to perform 45‐min recordings as a representative short time period considering both feasibility and the acquisition of sufficient rhythm assessment based on existing literature. The clinical application of SHR has several advantages. Short‐term recordings are performed in standardized conditions, eliminating the risk of ECG lead displacement, noise, and artifacts. Moreover, they are minimally influenced by external variables, such as patient activity, which increase intersubject variability. On the other hand, analysis of long‐term recordings can be costly, time‐consuming, and demanding, frequently requiring a thorough manual signal editing for reliable NIRFs results. Thus, the concept of a quick initial arrhythmic risk scanning of high‐risk patients, for less than an hour, looks attractive, and preferable. In this direction, a combined score of seven different cardiac risk stratifiers, mainly based on indices derived from controlled 30‐min ECG recordings in an undisturbed supine position, has been recently suggested as a risk stratification tool in both post‐MI patients and healthy individuals (Steger et al., 2019, 2021).

HRV reflects the overall state of the cardiac autonomic nervous system, analyzing the variation over time of consecutive heartbeat intervals. Both short‐ and long‐term recordings, ranging from 10 s to 24 h, have been used for the assessment of HRV, using time‐ and frequency‐domain methods (de Bruyne et al., 1999). HRV in short‐duration ECG recordings demonstrated a significant prognostic value in patients with heart failure (La Rovere et al., 2003). Fei et al. evaluated the predictive power of both short‐ and long‐term HRV in 700 patients with post‐MI and found that short‐term SDNN was significantly associated with cardiac mortality (Fei et al., 1996). In our study, we found that reduced SDNN in SHR is associated with a 4‐fold risk of PVS inducibility, albeit with moderate accuracy. This NIRF, in combination with other clinical and echocardiographic parameters, can be used as the first step of a practical risk stratification algorithm, which identifies truly high‐risk post‐MI patients (Bailey et al., 2001; Schmitt et al., 2001). These patients should be subsequently referred for further assessment with invasive PVS, to guide the final decision for ICD implantation.

According to current guidelines, the indication for ICD implantation for primary prevention is exclusively based on LVEF, with a cut‐off value of 35% (Al‐Khatib et al., 2018; Zeppenfeld et al., 2022). Our study verifies the significant role of LVEF as a risk indicator, even in patients with moderately reduced or preserved left ventricular systolic function; namely, LVEF in the range of 40%–50% was significantly associated with inducible PVS. This subgroup of patients, indeed, are at higher risk than those with LVEF >50%. A larger study focusing only on this population would potentially identify other ECG‐based risk markers that may yield significance. Moreover, patients in the high‐risk group had significantly increased LV dimensions compared with the non‐inducible group.

The results of the PRESERVE‐EF study indicate a 2‐step risk stratification approach, using both noninvasive arrhythmic risk markers and invasive electrophysiological study, for a more accurate risk assessment of post‐MI patients with LVEF ≥40%. This strategy identified finally a very high‐risk subgroup with an annual incidence of device activation of more than 8% that received major benefits from primary prevention ICD implantation. Moreover, none of the patients with undetectable risk factors or negative PVS eventually developed malignant arrhythmic events during follow‐up. Among NIRFs, LPs, and NSVT in 24‐h Holter ECG recordings demonstrated a significant prognostic value for positive PVS. LVEF <50% was also associated with PVS inducibility. The presence of the above 3 parameters was prognostic of positive PVS with a sensitivity of 98% (OR 14.15, *p* = .01) (Trachanas et al., 2022). We should note that LPs are usually measured in short‐duration ECG recordings, and could be added to a fast risk stratification approach with SHR. However, in the present analysis, LPs and NSVT were not significant predictors of PVS inducibility, potentially due to the limited sample size. Considering the significant prognostic role of NSVT detection in 24‐h ECG recordings, we can speculate that the identification of high‐risk patients with post‐MI may be improved with the application of longer duration ECG follow‐up (3‐day, 7‐day Holter, or even implantable loop recorder) or repetition of 24‐h recordings at regular time intervals. In this study, HRV, assessed in 24‐h ECG recordings with the predefined methodology and cut‐off values, failed to predict positive PVS. However, a trend toward reduced SDNN was observed in the positive PVS group, compared to the negative PVS group. We suspect that larger samples would be able to identify the prognostic value of HRV in long‐term ECG recordings, similar to our observations from SHR. Furthermore, the results would probably diverge if a different HRV method or normal cut‐off points had been selected.

The aforementioned data can be summarized in a multivariable risk assessment approach, which includes clinical characteristics (presence of diabetes, history of STEMI), echocardiography (LVEF), and SHR for the estimation of HRV and LPs. This initial screening tool should be combined with invasive PVS, as an integral second step, for the final decision on ICD implantation in high‐risk patients. The incorporation of advanced imaging modalities, such as cardiac magnetic resonance, may further improve the accuracy of this risk stratification strategy and is a subject of ongoing research (Dagres et al., 2020). As the sample size of the present study is limited, and the endpoint was defined as the inducibility of ventricular arrhythmias during PVS, this hypothesis‐generating study may set the stage for a more comprehensive understanding of the risk profile of patients with post‐MI.

## 5 LIMITATIONS

Our study is subject to the inherent problems of confounding and biases of observational studies. The sample size is small and may not be adequate to reveal further significant differences in NIRFs between the compared groups. There is a lack of a universally accepted consensus for the calculation, optimal recording duration, and normal cut‐off values for each electrocardiographic parameter. The selected methodologies have been previously described by experts and applied in large clinical studies, however, the use of other assessment modes may yield different results. The outcome was defined as the inducibility of malignant ventricular arrhythmias in PVS, and larger prospective studies with long‐term follow‐up for major clinical events should be sought for confirmation of our results. The electrocardiographic indices of autonomic function can be assessed only in sinus rhythm. Patients with permanent atrial fibrillation or paced rhythm were excluded from the study. Finally, age < 80 years was another inclusion criterion. Therefore, the results cannot be extrapolated to elderly patients.

## 6 CONCLUSIONS

Post‐MI patients with preserved LVEF are currently excluded from any type of SCD risk stratification. In these patients, 45‐min SHR provides important prognostic information, as reduced SDNN is a significant, independent predictor of PVS inducibility.

HRV assessed from SHR, combined with other noninvasive clinical and echocardiographic variables (diabetes, history of STEMI, LVEF), can form a useful, practical, and rapid screening risk assessment tool for general cardiologists. This approach can be widely implemented in everyday clinical practice since it can be performed even during a single outpatient clinic visit.

## 7 AUTHOR CONTRIBUTIONS

**Konstantinos Triantafyllou** contributed to the conceptualization, formal analysis, methodology, investigation, visualization, writing—original draft preparation, and writing—review and editing. **Nikolaos Fragakis** and **Vassilios Vassilikos** contributed to the conceptualization, formal analysis, methodology, supervision, visualization, writing–original draft preparation, and writing—review and editing. **Konstantinos A. Gatzoulis** contributed to the conceptualization, methodology, supervision, writing—original draft preparation, and writing—review and editing. **Antonios Antoniadis** and **Georgios Giannopoulos** contributed to the formal analysis, visualization, writing—original draft preparation, and writing—review and editing. **Petros Arsenos** contributed to the methodology, visualization, writing—original draft preparation, and writing—review and editing. **Dimitrios Tsiachris** contributed to the methodology, writing—original draft preparation, and writing—review and editing. **Christos‐Konstantinos Antoniou**, **Konstantinos Trachanas**, and **Konstantinos Tsimos** contributed to the investigation, writing—original draft preparation, and writing—review and editing.

## CONFLICT OF INTEREST STATEMENT

None.

## ETHICS STATEMENT

This study was approved by the Ethical Committee of the Aristotle University of Thessaloniki, Greece, and followed the ethical Declaration of Helsinki. Patients gave informed consent for their participation.

## Data Availability

The data that support the findings of this study are available from the corresponding author upon reasonable request.
